# Novel and Potential Photoprotective and Tyrosinase Inhibitory Effects of *Tetrastigma erubescens* Extracts: Evidence from In Vitro Assays and Computational Approach

**DOI:** 10.3390/life15070995

**Published:** 2025-06-22

**Authors:** Thi Thu Le Vu, Tu Quy Phan, Tien Lam Do, Van Bon Nguyen

**Affiliations:** 1 Department of Basic Science, Thai Nguyen University of Agriculture and Forestry (TNU), Quyet Thang, Thai Nguyen 24119, Vietnam; vuthithule@tuaf.edu.vn; 2 Department of Science and Technology, Tay Nguyen University, Buon Ma Thuot 630000, Vietnam; 3 Institute of Natural Products Chemistry, Vietnam Academy of Science and Technology, 18 Hoang Quoc Viet, Cau Giay, Hanoi 10072, Vietnam; dotienlam198@gmail.com; 4 Faculty of Chemistry, Graduate University of Science and Technology, Vietnam Academy of Science and Technology, 18 Hoang Quoc Viet, Cau Giay, Hanoi 10072, Vietnam; 5 Institute of Biotechnology and Environment, Tay Nguyen University, Buon Ma Thuot 630000, Vietnam

**Keywords:** *Tetrastigma erubescens*, UV protection, tyrosinase inhibitors, docking, DFT

## Abstract

*Tetrastigma erubescens*, a native medicinal plant of Vietnam, has long been used in folk medicine to manage various diseases, including skin-related issues. However, limited research has been conducted on this herb’s bioactivities and chemical composition. This study aims to investigate the chemical constituents and evaluate the anti-tyrosinase activity and UV-A/UV-B absorption capacity of *T. erubescens* extracts, highlighting their potential as natural sources for skin-whitening and sun protection agents. In vitro assays demonstrated that the ethyl acetate (EA) extract of *T. erubescens* exhibited a significant UV-A and UV-B absorption capacity. Notably, this extract showed a strong anti-tyrosinase activity for the first time, with a maximum inhibition rate of 99.2% and an IC_50_ value of 70.3 µg/mL. Based on the UHPLC and GCMS analysis, phenolic compounds (**1**–**9**) and ten volatile constituents (**10**–**19**) were identified in the EA extract of *T. erubescens*. Of these, almost all volatiles and some phenolics were reported for the first time in this genus. The molecular docking analysis revealed that all identified phytochemicals showed a comparable or greater binding affinity to both mushroom tyrosinase (docking scores: from −7.5 to −14.1 kcal/mol) and human tyrosinase (from −6.7 to −14.8 kcal/mol) than kojic acid (−8.7 and −8.6 kcal/mol, respectively). In addition, these identified compounds showed favorable drug-like properties and low toxicity risks via ADMET prediction and Lipinski’s Rule of Five analyses. The results obtained in this work suggest that the EA extract of *T. erubescens* is a promising natural source of bioactive compounds for cosmetic applications, particularly in whitening and sun protection formulations.

## 1. Introduction

Plant-derived compounds are extensively used in both traditional and modern healthcare systems for the prevention and treatment of various diseases worldwide, owing to their chemical diversity and biological activity [1,2]. Approximately 80% of the global population has used herbal products in healthcare, and 75% of Vietnamese people rely on traditional remedies derived from medicinal plants [2,3]. Vietnam ranks among the sixteen most biodiverse countries globally, with more than 10,000 plant species recorded. Among these, approximately 4000 are used in traditional medicine, and about 700 are listed in the Vietnamese pharmacopeia [3]. Plant-derived natural products, including polyphenols and flavonoids, have also been widely applied in cosmetics for sun protection, skin whitening, anti-aging, and the treatment of acne, eczema, and scars [4,5]. In this work, we explore the potential whitening and photoprotective effects of *Tetrastigma erubescens* Planch. extracts.

The genus *Tetrastigma* Planch. (Vitaceae) comprises approximately 100 species and is widely distributed across Asian countries and Oceania [6]. While many species are traditionally used in folk medicine, only a few have been scientifically studied for their phytochemical constituents and bioactivities [6,7]. Several species, including *T. hypoglaucum*, *T. planicaule*, *T. obtectum*, and *T. hemsleyanum*, have been extensively studied in China and documented in numerous publications. In contrast, research on the pharmacological and cosmetic potential of *T. erubescens* remains limited, with only a few reports originating from Vietnam [8].

*T. erubescens* is native to China, Laos, Cambodia, Thailand, and Vietnam. In Vietnam, it is commonly found in regions including Lao Cai, Vinh Phu, Thai Nguyen, Lam Dong, and Kon Tum [8]. Traditionally, this herb has been used as a folk remedy to treat stomachaches, fevers, hypertension, inflammation, and other conditions [8]. Despite its traditional use, scientific research on the phytochemical profile and bioactivities of *T. erubescens* remains scarce, with only a few studies available in the literature [8].

To further investigate the phytochemical composition and cosmetic potential of *T. erubescens*, the plant was collected in the Central Highland of Vietnam and extracted using various solvents. The resulting extracts were evaluated for their phytochemical profiles, anti-tyrosinase activity, and photoprotective properties. An ADMET analysis and Lipinski’s Rule of Five were applied to evaluate the drug-like properties of the identified phytocompounds. Additionally, molecular docking and DFT calculations were performed to investigate the interactions between active phytocompounds and the target enzyme tyrosinase.

## 2. Materials and Methods

### 2.1. Materials

The aerial parts of *Tetrastigma erubescens* Planch were collected from Dak Lak province, Vietnam, on 20 May 2023. The plant material was authenticated by Prof. Dr. Tran Bach (Institute of Biotechnology, Vietnam Academy of Science and Technology), and a voucher specimen (Code: TTH) was deposited at the Department of Science and Technology, Tay Nguyen University, Buon Ma Thuot 630000, Vietnam. After collection, the samples were cleaned, air-dried in the shade at ambient temperature (25–30 °C), and stored in polyethylene (PE) bags at −30 °C until further use. Mushroom tyrosinase (EC 1.14.18.1), kojic acid, L-DOPA, gallic acid, catechin, chlorogenic acid, EGCG, epicatechin gallate, vitexin, rutin, quercetin, and apigenin were obtained from Sigma-Aldrich (St. Louis, MO, USA).

All solvents and reagents used were of analytical or HPLC grade.

### 2.2. Preparation of T. erubescens Extract

The extract preparation followed a modified protocol based on our previous work [9]. Dried aerial parts of *T. erubescens* (10 g) were separately extracted as portions with 100 mL of hexane, methanol, and ethyl acetate. Each extraction was performed under continuous shaking at 60 °C for 24 h. After filtration through Whatman No. 1 filter paper, the plant residue was re-extracted twice under the same conditions using fresh portions of solvent. The combined filtrates for each solvent were concentrated under reduced pressure at 60 °C using a rotary evaporator. The dried extracts were stored at −30 °C until further analysis.

### 2.3. Identification of Phytocompounds of T. erubescens Extract

Gas Chromatography–Mass Spectrometry (GCMS) analysis was performed to identify volatile compounds according to the protocols in our previous report [10]. The herbal extract was diluted in methanol and filtered via solid-phase QuEChERS extraction. A Gas Chromatography system (Trace GC Ultra, Thermo Fisher Scientific, Waltham, MA, USA) coupled with an ITQ900 mass spectrometer (Thermo Fisher Scientific, Waltham, MA, USA) was applied for analysis. A capillary column TG-SQC (30 m × 0.25 mm × 0.25 μm) was used. Pure Helium was used as the carrier gas (99.999%) at a flow rate of 1 mL/min. A 1 μL extraction solution was injected into the GCMS system with a split ratio of 10:1. The ion source temperature and injector temperature were maintained at 230 and 250 °C, respectively. The temperature program of the oven started at 70 °C (2 min), then increased to 280 °C with a rate of 15 °C per minute. The MS data were acquired at 70 eV, a scan interval of 0.5 s, and a fragment range from 50 to 650 Da. Volatiles were identified via comparing with the reference compound data from the NIST 17. L and Wiley Mass Spectra Libraries.

High-performance liquid chromatography (HPLC) was conducted to analyze phenolic compounds in the herbal extract following the protocol presented in the previous report [11]. The herbal extract was dissolved at 10 mg/mL concentration in MeOH and filtered via membrane PVDF filter at size of 0.45 μm (MilliporeSigma, Burlington, MA, USA). Then, 2 μL of the herbal solution was injected into the HPLC system (Thermo Ultimate 3000) with a column (3 μm, 150 × 2.1 mm; Hypersil GOLD aQ). MeOH in 0.1% phosphoric acid was used as the mobile phase, with an increasing gradient beginning at 5% to 95% MeOH. The detailed program was as follows: 0.0–0.5 min (5% MeOH), 0.5–8.0 min (5–30% MeOH), 8.0–13.0 min (30–45% MeOH), 13.0–18.0 min (45–65% MeOH), 18.0–22.0 min (65–95% MeOH), and 22.0–23.0 min (95–5% MeOH). A mobile phase flow rate was set at 0.2 mL/min of flow rate, and a temperature of 30 °C was maintained for the column. The detection wavelength was set at 265 nm. The content of phenolics compounds was calculated using below equations:

**Gallic acid**: y = 0.2561C_GA_ + 0.005, R^2^ = 0.9998

**Catechin**: y = 0.0294C_Cat_ + 0.0024, R^2^ = 0.9999

**Chlorogenic acid**: y = 0.0561C_CGA_ − 0.0019, R^2^ = 0.9997

**EGCG**: y = 0.087C_EGCG_ − 0.0374, R^2^ = 0.9993

**Epicatechin gallate:** y = 0.3824C_ECG_ − 0.0037, R^2^ = 0.9999

**Vitexin:** y = 0.1376C_Vit_ − 0.0068, R^2^ = 0.9999

**Rutin:** y = 0.5559C_Rut_ + 0.1448, R^2^ = 0.9997

**Quercetin:** y = 0.2232C_Quer_ + 0.2076, R^2^ = 0.9999

**Apigenin:** y = 0.2354C_Api_ − 0.0682, R^2^ = 0.9994.

### 2.4. Tyrosinase Inhibition and Ultraviolet Radiation Absorption Efficiency Assays

*Tyrosinase inhibition assay:* The inhibitory activity against tyrosinase was evaluated using the protocol previously presented by Deng et al. [12]. The herbal extracts, enzyme tyrosinase, and L-dopa were dissolved in sodium phosphate buffer (0.05 M, pH 6.8). Then, 100 μL of sample solution was mixed with 100 μL of mushroom tyrosinase in a 96-well plate and incubated at 25 °C for 5 min, and then 100 μL of L-dopa was added to initiate the reaction, which continued for another 5 min at 25 °C. The final mixture of the reaction solution was measured at the wavelength of 475 nm to determine the dopachrome content in the reaction solution. Kojic acid, a commercial tyrosinase inhibitor, was also used as a positive control. The enzyme inhibition (%) was calculated using the following equation:

Tyrosinase inhibition (%) = [(C − E)/C] × 100,

where E is the absorption value of experimental wells containing a mixture solution with the presence of tyrosinase, herbal extracts, and L-dopa, while C is the absorption value of controlling wells containing a mixture solution with the presence of tyrosinase and L-dopa but without herbal extracts. The IC_50_ value was also calculated.

*Ultraviolet radiation absorption efficiency assay*: The UV absorption efficiency of the *T. erubescens* extracts was evaluated following the method described by Seregheti et al. [13], with minor modifications. Briefly, the absorbance of each extract (dissolved in ethanol, 100 µg/mL) was measured at wavelengths ranging from 200 to 400 nm using a UV–Vis spectrophotometer. The photoprotective potential was determined based on the absorbance peaks within the UV-A (320–400 nm) and UV-B (280–320 nm) ranges.

### 2.5. Computational Study

*Docking study:* Molecular docking simulations were performed according to the typical steps presented in some previous works [11,14,15]. The protein structures (tyrosinase, 2Y9X, and 7RK7) were obtained from the RCSB Protein Data Bank. Their 3D structures and the active binding sites (BSs) were prepared by MOE-2015.10 software. The preparations were conducted at a virtual pH of 7.0. The ligand structures (phytocompounds identified from the *T. erubescens* extract and kojic acid) were prepared using ChemBioOffice 2018 and MOE software. Some major parameters were set as follows: force field MMFF94x; dielectric constant (R-Field) set to 80; cell angles = 90°; gradient set to 0.01 RMS kcal·mol^−1^·Å^−2^; pH adjusted to 7.0; and additional settings included a cutoff, rigid water molecules, space group P1, and a simulation cell size of 10 × 10 × 10 Å^3^. The prepared ligands were docked into the binding sites on protein 2y9x using the same software MOE. Root-mean-square deviation (RMSD), docking score (DS), interaction types, distances between linkages, and amino acid compositions were harvested as key output data for analysis.

*DFT calculation:* The frontier molecular orbitals—highest occupied molecular orbital (HOMO) and lowest unoccupied molecular orbital (LUMO)—properties of the major compounds were examined using density functional theory (DFT) at the B3LYP/6-31G theoretical level.

The drug-likeness and pharmacokinetic properties of the phytocompounds were investigated based on Lipinski’s Rule of Five (LR5) and pharmacokinetic parameters, including absorption, distribution, metabolism, excretion, and toxicity (ADMET) parameter analysis. LR5 analysis was conducted using an online tool at https://scfbio-iitd.res.in/Sanjeevini/Lipinski.php (accessed on 9 December 2024). ADMET was assessed using the SwissADME web tool. Key output parameters—including water solubility, intestinal absorption, BBB permeability, CYP enzyme interactions, and toxicity profiles (e.g., AMES toxicity, hERG inhibition, and hepatotoxicity)—were analyzed and interpreted according to established guidelines reported by Dulsat et al. [16]. Details and data on the pharmacokinetic parameters were obtained from a public repository at http://biosig.unimelb.edu.au/pkcsm/theory (accessed on 10 December 2024).

## 3. Results and Discussion

### 3.1. Evaluating Tyrosinase Inhibitory and Photoprotective Effect of T. erubescens Extract

#### 3.1.1. Photoprotective Effect of *T. erubescens* Extracts

**Figure 1:**
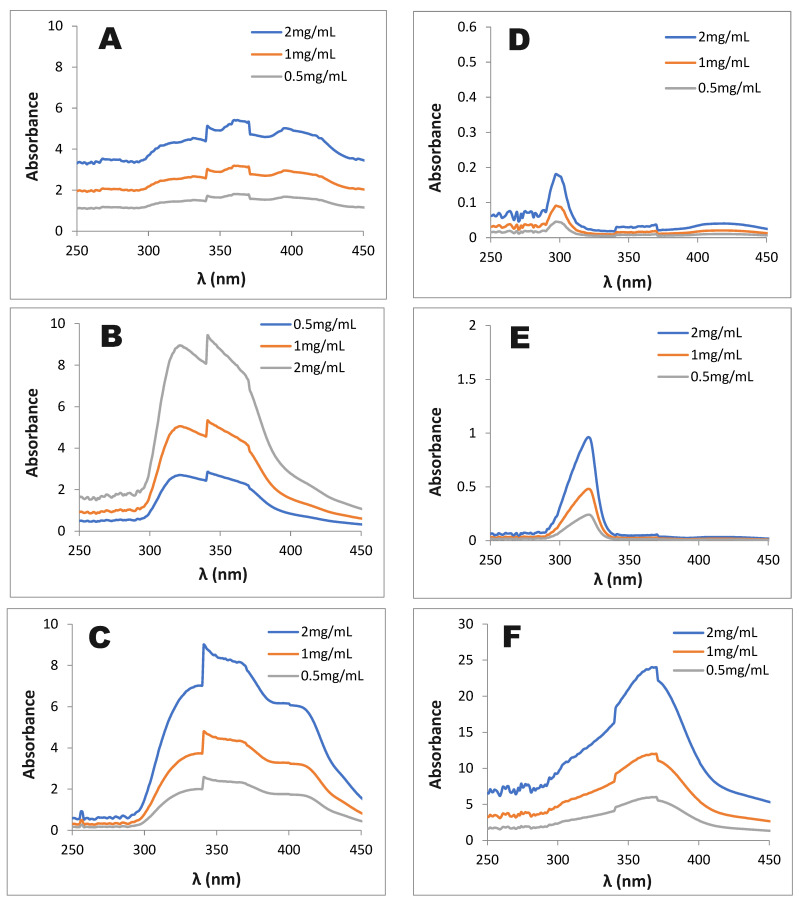
The UV absorption efficiency of the hexane extract (**A**), MeOH extract (**B**), and ethyl acetate extract (**C**) of *T. erubescens* and some commercial compounds, including gallic acid (**D**), vitamin C (**E**), and quercetin (**F**). The extracts at 0.5, 1, and 2 mg/mL concentrations were detected in the UV/vis spectrum at wavelengths from 250 to 450 nm.

Solar ultraviolet (UV) radiation has been shown to display many harmful effects on human health, including erythema, hyperpigmentation, sunburn, inflammation, wrinkling, photoaging, hyperplasia, local immunosuppression, and skin photo-carcinogenesis [17,18]. UV radiation is classified into three major categories: UV-C, UV-B, and UV-A, with wavelengths in the line of 100–280, 280–315, and 315–400 nm, respectively [18]. The ozone layer completely absorbs UV-C and almost all of UV-B. Thus, it has been recorded that the sun’s UV radiation reaches the earth’s surface with 95% UV-A and 5% UV-B. In addition, the ozone layer has been destroyed, and as such, it increases the UV-B reaching the earth’s surface [18]. Thus, the anti-UV-A and anti-UV-B radiation have been suggested as important values for investigating sun protection agents. The experimental data are recorded and illustrated in Figure 1.

As shown in Figure 1, all the solvent extracts showed a positive UV-A and UV-B absorption. Of these, the hexane extract (Figure 1A) has a moderate absorption of both UV-A and UV-B radiation, while the MeOH extract (Figure 1B) and ethyl acetate extract (Figure 1C) display a high absorption efficiency against UV-A and UV-B radiation. For comparison, some commercial compounds, including gallic acid, quercetin, and vitamin C, were also evaluated against UV-A and UV-B radiation. As shown in Figure 1D,E, both the garlic acid and vitamin C showed no absorption of UV-A, while they displayed minor and moderate absorption efficacies against UV-B radiation, respectively. In contrast, quercetin showed a high UV-A and UV-B radiation absorption efficacy. The result indicated that *T. erubescens* extracts shower a higher absorption of UV-A and UV-B radiation than that of garlic acid and vitamin C, while they demonstrated a weaker absorption efficacy against UV-A and UV-B radiation than that of quercetin. Concerning the UV-C absorption, the hexane extract showed the most efficacy (Figure 1A), followed by the MeOH extract (Figure 1B), while the EA extract displayed a weak UV-C absorption (Figure 1C). Among commercial compounds, quercetin also demonstrated the highest UV-C absorption (Figure 1F). In the comparison, the hexane extract showed a UV-C absorption comparable to quercetin.

All the herbal extracts and quercetin show the efficacy of the UV absorption at the wavelength in the range of 290–320 nm; as such, these samples were further investigated for an in vitro determination of the sun protection factor (SPF) following the method previously presented by Seregheti et al. [13]. The MeOH extract, EA extract, and quercetin showed high SPF values of 8.32, 7.25, and 6.21, respectively, while the hexane extract demonstrated a lower efficacy with an SPF value of 4.85 at the tested concentration of 0.1 mg/mL. Natural products with SPF values greater than 6.0 are considered potential sunscreens. In this study, the MeOH and EA extracts of *T. erubescens* showed high SPF values (7.25–8.32); thus, these herbal extracts may be potential candidates for sun protection factors.

#### 3.1.2. Tyrosinase Inhibitory Activity of *T. erubescens* Extracts

**Figure 2:**
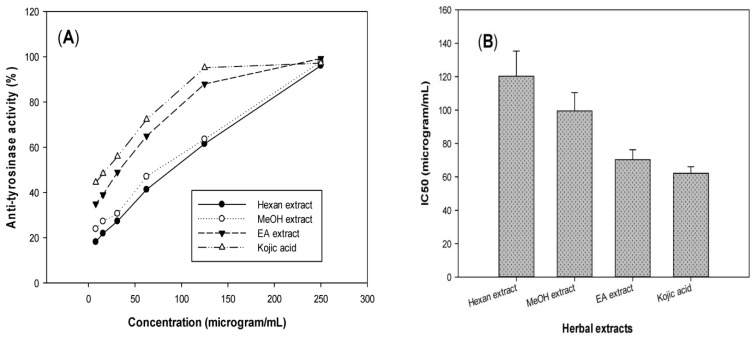
Tyrosinase inhibitory activity of different solvent extracts of *T. erubescens* with inhibition (%) value (**A**) and IC_50_ value (**B**).

Tyrosinase, a key enzyme, catalyzes the first and only rate-limiting step in melanogenesis; as such, the inhibition of this enzyme results in skin whitening and an anti-melanogenic effect [19]. For evaluating the potential anti-tyrosinase activity of *T. erubescens*, this herbal sample was extracted with several solvents, such as hexane, ethyl acetate (EA), and MeOH, then used for testing. As shown in Figure 2, the *T. erubescens* EA extract demonstrated the most potent activity with maximum inhibition and IC_50_ values of 99.2% and 70.3 μg/mL, respectively. Kojic acid, a commercial tyrosinase inhibitor, was also tested in the same condition for comparison and exhibited the highest inhibitory effect, with a maximum inhibition and IC_50_ value of 97% and 62.1 μg/mL, respectively. This result indicated that the *T. erubescens* EA extract shows a potential anti-tyrosinase activity, which is comparable to that of kojic acid.

Several species within the genus *Tetrastigma* have been traditionally used in folk medicine to treat a variety of diseases [7]. These species have been reported to exhibit diverse pharmacological activities, including antioxidant, antitumor, antiviral, hepatoprotective, anti-inflammatory, and analgesic effects [7]. Additionally, anti-diabetic properties have been observed through the modulation of glucokinase, AMP-activated protein kinase, glucose-6-phosphatase, and phosphoenolpyruvate carboxykinase expression [20]. In the context of enzyme inhibition for drug discovery, certain *Tetrastigma* species have demonstrated an inhibitory activity against epoxide hydrolase and nitric oxide synthase [21]. *Tetrastigma erubescens* is widely used in traditional medicine for treating stomachaches, fevers, inflammation, hypertension, and other ailments in Vietnam and neighboring regions. However, only limited studies have been conducted on the drug discovery potential of extracts from this species [8]. Therefore, the findings of the present study may contribute to expanding the current knowledge on the bioactivities of this medicinal plant.

### 3.2. The Phytochemical Profile of the T. erubescens Ethyl Acetate Extract

Among the various solvent extracts tested, the ethyl acetate (EA) extract exhibited the highest anti-tyrosinase activity and a strong absorption capacity in both UV-A and UV-B regions. Therefore, this extract was selected for a further phytochemical characterization using UHPLC and GC-MS analyses. As summarized in Table 1, the UHPLC analysis identified nine phenolic compounds, including two polyphenols and seven flavonoids. Among these, gallic acid (**1**) was the predominant constituent in the *T. erubescens* EA extract, with a concentration of 30.837 μg/mg of the dried herbal extract. Catechin (**2**), chlorogenic acid (**3**), epicatechin gallate (**5**), and rutin (**7**) were also detected at moderate levels, ranging from 1.284 to 2.793 μg/mg. The remaining compounds—EGCG (**4**), vitexin (**6**), quercetin (**8**), and apigenin (**9**)—were present in lower concentrations.

As shown in Table 2, the GC–MS analysis identified ten volatile compounds (compounds **10**–**19**). Of these, 2H-Pyran-2-carboxylic acid, 3,6-dihydro-6-propoxy-, ethyl (**18**) were found to be the most abundant contents in the sample, with an area percentage of 60.91% of the area; compounds **14**, **16**, **17**, and **19** were detected with moderate area percentages in the range of 5.85–10.5%; while other volatile compounds (**10**, **11**, **12**, **13**, and **15**) were found in the herbal extract in minor amounts (≤1.42% of area). The UHPLC and GC–MS fingerprints are provided in Figures S1–S12 (Supplementary Materials).

According to Zhang et al. (2022) [7], more than 240 phytocompounds have been identified from various *Tetrastigma* species, including 74 flavonoids, 21 phenylpropanoids, 19 steroids, 14 terpenoids, 14 alkaloids, and other phytochemicals. Most of these studies have focused on *T. hemsleyanum*, *T. hypoglaucum*, *T. obtectum*, and *T. planicaule*. In contrast, the phytochemical profile of *T. erubescens* remains relatively unexplored. To date, only one study by Dao et al. (2014) [8] has reported the isolation of nineteen phytocompounds from the stem extract of *T. erubescens*, including tetrastigmol A, seven flavonoids, two steroids, bergenin and its derivative, three stilbenes, lignin, a benzenecarboxylic acid derivative, and two norisoprenoids. In the present study, all nine phenolic compounds detected and identified in the ethyl acetate (EA) extract of *T. erubescens* have been previously reported in other species of the genus, supporting the conserved nature of certain phytochemicals across *Tetrastigma*. Notably, the ten volatile compounds identified in this study have not been previously documented in any other species of the genus *Tetrastigma*. This highlights a novel aspect of the chemical profile of *T. erubescens* and contributes new information to the phytochemical diversity of this medicinal plant.

Several flavonoids identified in the *T. erubescens* EA extract—namely apigenin, vitexin, quercetin, and catechin—have been widely reported as tyrosinase inhibitors. Apigenin exhibits a moderate inhibitory activity through the chelation of copper ions at the enzyme’s active site, which is supported by both biochemical assays and molecular docking studies [22,23]. Vitexin, though structurally similar, shows a weaker inhibition, possibly due to its glycosidic substitution pattern [22]. Quercetin acts as a competitive inhibitor of tyrosinase, inducing conformational changes and blocking the access to the catalytic core [24,25]. Catechin and its derivatives also inhibit tyrosinase via direct binding and oxidative interaction mechanisms [26,27]. These previous findings support the observed anti-tyrosinase potential of the EA extract and support its application as a natural melanogenesis modulator.

### 3.3. Molecular Docking, DFT Calculation, Drug-Likeness, and Pharmacokinetic Parameters of Major Phytocompounds

#### 3.3.1. Molecular Docking Study and DFT Calculation

**Figure A1:**
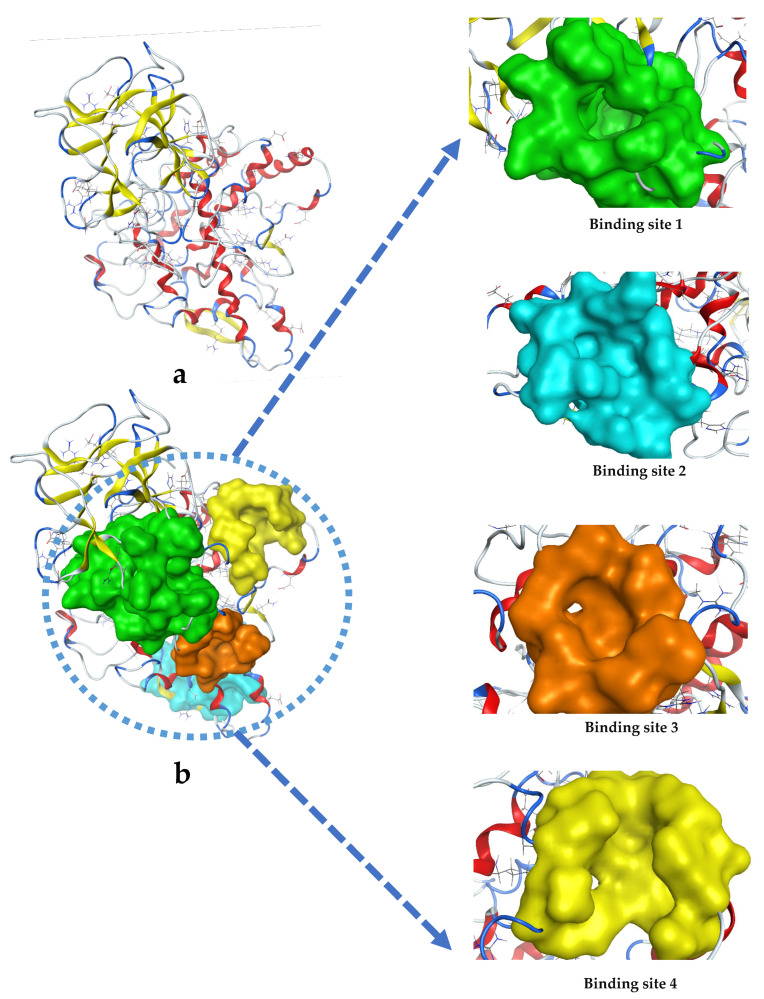
(**a**) The three-dimensional structure of tyrosinase (PDB ID: 2Y9X). (**b**) A visualization of four predicted ligand binding sites on the enzyme surface.

**Figure A2:**
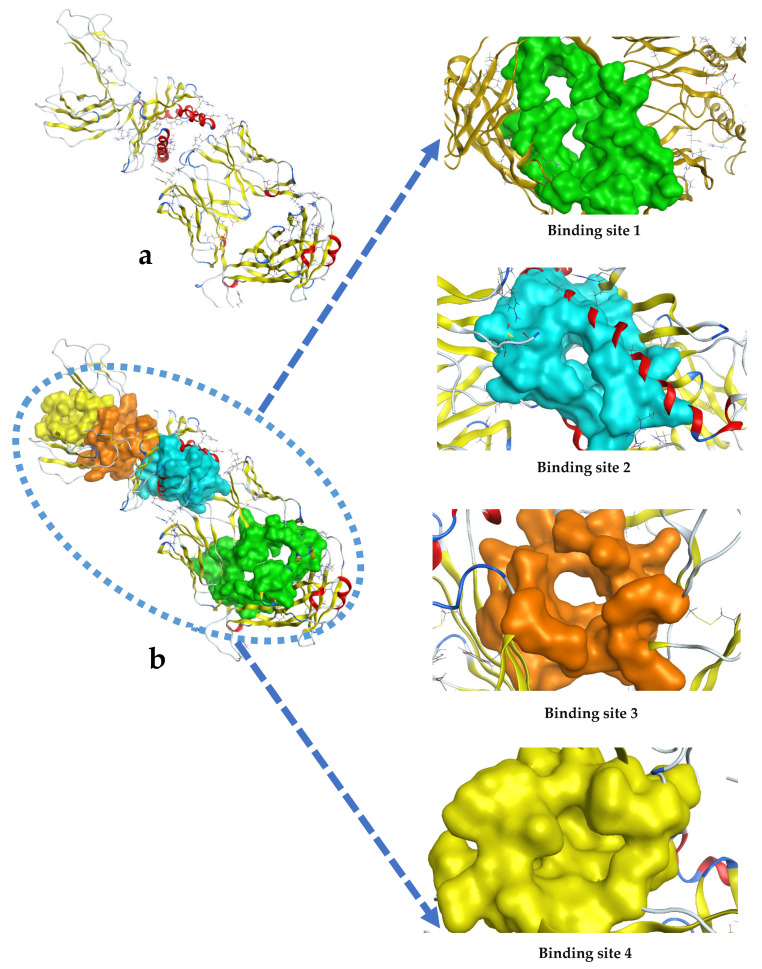
(**a**) Three-dimensional structure of tyrosinase (PDB ID: 7RK7). (**b**) A visualization of four predicted ligand binding sites on the enzyme surface.

The molecular docking was performed according to the protocols presented in some previous reports using MOE software [11,14,15]. The crystal structure of the mushroom tyrosinase (PDB ID: 2Y9X) was retrieved from the RCSB Protein Data Bank. Binding sites (BSs) were predicted using the ‘Site Finder’ function in MOE. As shown in Figure A1 and Figure A2 in the Appendix A section, four BSs were detected on protein 2Y9X and protein 7RK7. All the ligands, including nineteen phytocompounds identified from the *T. erubescens* EA extract in this study, and kojic acid (a commercial tyrosinase inhibitor) were docked into all these four BSs, and for each ligand, only the best-scoring interaction (lowest docking score) was recorded and analyzed in detail [11]. The detailed information, such as the sizes and residues in these binding sites on protein 2Y9X and 7RK7, is shown in Table A1 and Table A2 in the Appendix B section, respectively.

In docking studies, two key parameters—the RMSD and docking score (DS)—are commonly used to assess the validity and strength of the ligand–receptor binding. Successful docking is typically indicated by an RMSD ≤ 2.0 Å, and effective binding is indicated by a DS ≤ −3.20 kcal/mol [28]. As shown in Table 3, all the docked ligands have RMSD values in the range of 0.93–1.90 Å; these results indicated that all the tested ligands successfully interact with the protein 2Y9X. Concerning effective binding, these compounds show the low DS values from −7.5 to −14.1 Kcal/mol, which is a comparable or more effective energy binding than that of kojic acid (DS value of −8.7 Kcal/mol), indicating a strong binding affinity of these phytocompounds toward the binding sites of 2Y9X. Of these ligand compounds, seven phenolic compounds (**2**,**4**–**8**) demonstrated a potential binding energy to 2Y9X with very low DS values (lower than −13.1 Kcal/mol), while almost all volatiles showed weaker interactions with protein 2Y9X, with DS values in the range from −7.5 to −12.6 Kcal/mol. In the DS value comparison, the binding efficacy of these ligands toward the protein 2Y9X was in the following range: **15** < **16** < **17** < kojic acid < **13** < **18** < **14** < **12** <**11** < **19** < **10** ≤ **1** < **9** < **3** < **2** <**5** ≤ **6** < **7** < **4** < **8.** Regarding the potential binding of these compounds against the human tyrosinase 7RK7, the docking study was also performed. As shown in Table 3, almost all the phenolic compounds demonstrated a higher binding energy than the volatile compounds. Of the identified compounds from the *T. erubescens* ethyl acetate extract, compounds **4**, **5**, and **9** were highly bound to 7RK7, with an effective energy binding in the range from −12.4 to −14.8 Kcal/mol, which is much lower than that of the commercial inhibitor kojic acid (−8.6 Kcal/mol). In the DS value comparison, the binding efficacy of the above compounds toward the protein 7RK7 was in the following range: **17** < **16** ≤ **18** < **14** < **12** < **11** ≤ **15** ≤ **19**≤ kojic acid < **1** < **13** < **7** < **10** < **3** < **2** ≤ **8** < **6** < **5** < **9** < **4**. This DS value ranking indicated that almost all the phytocompounds showed a comparable or higher binding interaction energy towards the proteins 2Y9X and 7RK7 with that of the commercial tyrosinase inhibitor.

**Figure 3:**
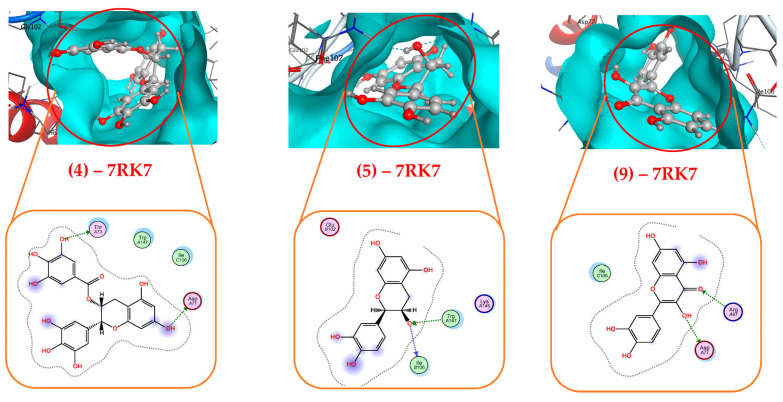
Inside the interactions of the most active phytocompounds (compounds **4**, **5**, and **9**) of the *T. erubescens* EA extract with tyrosinase (7RK7) at binding site 2 via the docking simulation.

**Figure 4:**
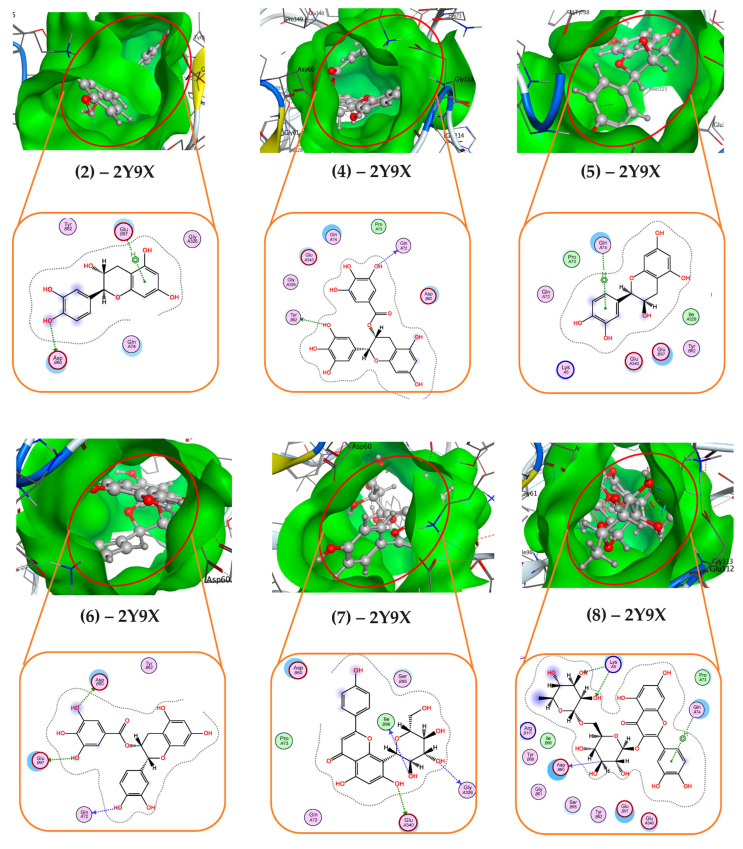
Inside the interactions of the most active phytocompounds (compounds **2**, **4**–**8**) of the *T. erubescens* EA extract with tyrosinase (2Y9X) at binding site 1 via the docking simulation.

Some of the most active phytocompounds (**4**, **5**, **9**) were further checked inside their interaction with the target protein 7RK7 (Table 3 and Figure 3). Ligand **4** effectively interacted with protein 7RK7 via connecting with amino acids Asp77 and Thr73 to form two H-donor linkages. Ligand **5** was also found binding to 7RK7 via creating two linkages (H-donor and H-acceptor) with amino acids Ile106 and Trp147, respectively, while ligand **9** interacted with Asp77 and Arg97, forming three bonds—one H-donor and two H-acceptor linkages. Regarding the interaction with protein 2Y9X, some of the most active phenolics (compounds **2**,**4**–**8**) were further checked inside their interaction with the target protein. The detailed binding at BS1 is recorded and presented in Table 4 and Figure 4. All these ligands were found to effectively bind to protein 2Y9X at BS1 by forming 2–3 linkages. Of these, ligand **7** was also found binding to 2Y9X at BS1 by creating up to four linkages, including 1 H-donor, 2 H-acceptors, and 1 pi-H by interacting with some amino acids of Asp60, Lys5, Lys5, and Gln74, respectively. Ligands **5** and **6** bound to 2Y9X by forming three H-donor linkages by interacting with amino acids Gln72, Glu97, and Asp60 (for ligand **5**) and Ile96, Gly326, and Glu340 (for ligand **6**). Other ligands were found binding to 2Y9X by forming two linkages. Of these ligands, ligand **2** created one H-donor and one pi-H linkage by interacting with Tyr62 and Gln72, and ligand **8** formed one H-donor and one H-acceptor by binding to Asp60 and Leu75.

So far, some phenolic compounds have been investigated for their interactions targeting enzyme tyrosinase, such as apigenin and vitexin quercetin, and garlic acid was extensively analyzed in the molecular docking study. However, other phenolic and especially volatile compounds, including the ten volatile constituents identified from the *Tetrastigma erubescens* extract in this study, have rarely been explored in docking analyses [24,29,30]. In this study, we extensively investigated the interactions of these identified compounds in both the tyrosinase originating from the mushrooms and humans. Furthermore, density functional theory (DFT) calculations, drug-likeness evaluations, and ADMET predictions were also conducted on these phytocompounds.

**Figure 5:**
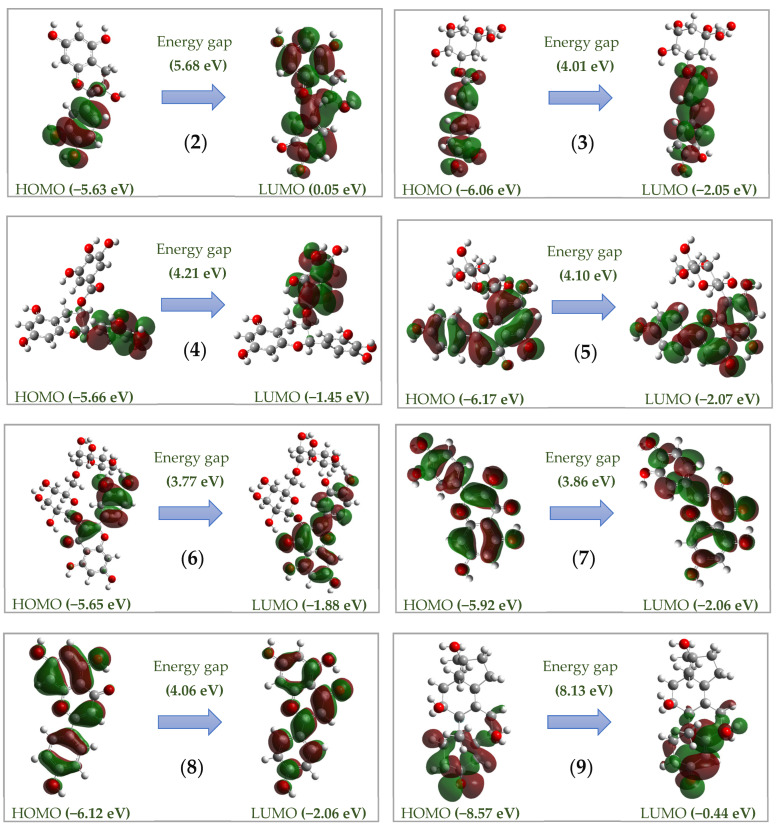
HOMOs and LUMOs of the most active ligands (**2**–**9**) and kojic acid (**20**), calculated using the DFT at the B3LYP/6-31G level of theory.

For elucidating the binding stability of the ligands, the highest occupied molecular orbital (HOMO) and the lowest unoccupied molecular orbital (LUMO) of these phytocompounds and commercial tyrosinase inhibitors were calculated and are presented in Figure 5. The structures of these ligands have low E_HOMO_ values (from −8.57 to −5.63 eV), indicating they have a significant electronic stability. According to Chen et al. (2022) [31], compounds with E_HOMO_ values below −5 eV are generally considered electronically stable. Furthermore, a ligand is regarded as having a favorable intermolecular binding potential with the target protein if its energy gap lies within the semiconducting range from 3.2 to 9.0 eV [32]. In this work, all the ligands showed their energy gap in the range from 3.86 to 8.13 eV; as such, they have a potential intermolecular binding capability with the targeting protein.

#### 3.3.2. Drug-Likeness and Pharmacokinetic Parameters of Phytocompounds

All the phytocompounds identified from the *T. erubescens* EA extract were further evaluated for their drug-likeness properties via the analysis of Lipinski’s Rule of Five (LR5). This rule’s content includes that a compound is more likely to possess drug-like properties if it meets the following criteria: a molecular weight ≤ 500 Da, a high lipophilicity (LogP value ≤ 5), possesses ≤ 5 hydrogen bond donors and ≤10 hydrogen bond acceptors, and its molar refractivity is in the range of 40–130. When a compound can satisfy more than two rules, it may be considered to possess good drug-likeness properties [33]. As shown in Table 5, compound **7** satisfies only three rules, and both compound **4** and compound **14** satisfy up to four rules, while other compounds (**1**–**3, 5**–**6, 8**–**13, 15**–**19**) satisfy all the rules of LR5. These results suggest that most phytocompounds from the EA extract exhibit favorable drug-likeness properties, implying a high potential for the further development as drug candidates.

ADMET (absorption, distribution, metabolism, excretion, and toxicity) profiles of these compounds were also predicted and are summarized in Tables S1 and S2 (Supplementary Data). In general, the ADMET predictions showed favorable outcomes, suggesting that most phytocompounds possess acceptable pharmacokinetic and safety profiles within standard thresholds.

Several compounds have been previously reported to exhibit a selective cytotoxicity toward cancer cells while sparing normal cells. For instance, EGCG was shown to inhibit HuCC-T1 cancer cells with minimal toxicity to 293T normal cells [34] and to reduce the WI38 normal cell viability by only 1% at 40–200 µM concentrations [35]. Chlorogenic acid also demonstrated a selective cytotoxicity towards cancer cells with limited effects on normal cells [36]. Similarly, epicatechin and epicatechin gallate exhibited anti-cancer activity without significant harm to normal cells [37,38]. Other compounds, including vitexin, apigenin, myricetin, and quercetin, showed a low or negligible toxicity toward normal cells [39,40,41]. Rutin, apigenin, and myricetin have also been identified as safe, effective adjuvant chemotherapeutic agents with minimal side effects, based on in vivo and clinical studies [42,43]. In summary, the ADMET prediction analysis indicates that most identified compounds from the *T. erubescens* EA extract are likely safe and non-toxic for human use. Nevertheless, these in silico predictions must be validated through comprehensive in vitro, in vivo, and clinical studies to confirm their therapeutic potential and safety profiles.

## 4. Conclusions

Among various solvent extracts of *T. erubescens*, the EA extract was found to show the efficiency of UV-A and UV-B absorption and a novel tyrosinase inhibitory effect for the first time. A total of nineteen phytocompounds were identified from the EA extract using UHPLC and GC-MS analyses. Notably, most of these compounds were newly detected in *T. erubescens*. In silico molecular docking studies revealed that nearly all identified compounds demonstrated favorable binding interactions with tyrosinase enzymes from both mushroom and human sources, showing promising binding affinities. ADMET predictions further suggested that the majority of these phytocompounds are likely to be non-toxic and possess drug-like properties. However, these findings are predictive; further in vitro, in vivo, and clinical studies are essential to confirm their safety and potential applications. The results obtained in this work suggested that *T. erubescens*, the EA extract, is a potential and rich source for the requirement of natural compounds with a potent tyrosinase inhibition and UV-protective potential.

## Figures and Tables

**Table 1:** Phenolic compounds identified and quantified by UHPLC in the EA extract of *Tetrastigma erubescens*.

No.	Name	Family	Retention Time (min)	Content (μg/mg of EA Extract)
**1**	Gallic acid	Non-flavonoid phenolic	4.135	30.837
**2**	Catechin	Flavonoid (flavan-3-ol)	12.157	2.454
**3**	Chlorogenic acid	Non-flavonoid phenolic	13.798	2.793
**4**	Epigallocatechin gallate (EGCG)	Flavonoid (flavan-3-ol)	14.388	0.835
**5**	Epicatechin gallate	Flavonoid (flavan-3-ol)	16.192	1.453
**6**	Vitexin	Flavonoid (C-glycosyl flavone)	17.643	0.168
**7**	Rutin	Flavonoid (glycoside)	19.883	1.284
**8**	Quercetin	Flavonoid (flavonol)	22.255	0.231
**9**	Apigenin	Flavonoid (flavone)	24.592	0.096

**Table 2:** Volatile compound profile of *Tetrastigma erubescens* ethyl acetate extract identified by GC/MS analysis.

No.	Name	Retention Time (min)	EA Extract
**10**	6β-Hydroxyfluoxymesterone	4.38	0.56
**11**	Paclobutrazol	5.26	0.68
**12**	Iso-olomoucine	7.98	1.4
**13**	α-Methylaminohexanophenone	19.72	1.42
**14**	Hexadecanoic acid, methyl ester (CAS)	28.42	10.39
**15**	Cyclohexane, 1,5-diethenyl-2,3-dimethyl-, (1α,2α,3α,5α)-	31.29	1
**16**	5-Decene, 4-ethynyl-, (E)- (CAS)	31.41	5.85
**17**	Cyclopentaneethanol, 2-(hydroxymethyl)-β,3-dimethyl- (CAS)	31.59	10.5
**18**	2H-Pyran-2-carboxylic acid, 3,6-dihydro-6-propoxy-, ethyl	38.69	60.91
**19**	Glafenin	39.62	7.28

**Table 3:** The interaction ability of the phytochemicals (ligands **1**–**19**) identified from *T. erubescens* and kojic acid (ligand **20**) with the mushroom tyrosinase (2Y9X) and human tyrosinase (7RK7) via the docking simulation.

The Interaction of Ligands (L) with 2Y9X				The Interaction of Ligands (L) with 7RK7			
Symbol of L-2Y9X	Docking Site	DS (Kcal/mol)	RMSD (Å)	Symbol of L-7RK7	Docking Site	DS (Kcal/mol)	RMSD (Å)
(**1**)—2Y9X	1	−11.3	1.128	(**1**)—7RK7	1	−9.7	1.21
(**2**)—2Y9X	1	−13.1	0.93	(**2**)—7RK7	2	−11.4	1.83
(**3**)—2Y9X	1	−12.6	1.30	(**3**)—7RK7	2	−10.9	0.92
(**4**)—2Y9X	1	−13.9	1.90	(**4**)—7RK7	2	−14.8	1.81
(**5**)—2Y9X	1	−13.5	1.51	(**5**)—7RK7	2	−12.4	1.53
(**6**)—2Y9X	1	−13.5	1.27	(**6**)—7RK7	1	−11.8	1.73
(**7**)—2Y9X	1	−13.7	0.98	(**7**)—7RK7	2	−10.5	1.38
(**8**)—2Y9X	1	−14.1	1.04	(**8**)—7RK7	2	−11.4	1.75
(**9**)—2Y9X	1	−12.1	1.01	(**9**)—7RK7	2	−12.5	1.44
(**10**)—2Y9X	1	−11.3	1.24	(**10**)—7RK7	1	−10.7	1.25
(**11**)—2Y9X	1	−10.9	1.78	(**11**)—7RK7	1	−8.6	1.07
(**12**)—2Y9X	1	−10.1	1.17	(**12**)—7RK7	1	−8.3	1.47
(**13**)—2Y9X	1	−8.9	1.51	(**13**)—7RK7	2	−10.4	1.38
(**14**)—2Y9X	1	−9.8	1.85	(**14**)—7RK7	1	−8.0	0.97
(**15**)—2Y9X	1	−7.5	1.17	(**15**)—7RK7	1	−8.6	2.00
(**16**)—2Y9X	1	−7.6	1.59	(**16**)—7RK7	2	−7.2	1.04
(**17**)—2Y9X	2	−8.5	1.79	(**17**)—7RK7	1	−6.7	1.14
(**18**)—2Y9X	1	−9.5	1.78	(**18**)—7RK7	1	−7.2	1.16
(**19**)—2Y9X	1	−11.1	1.66	(**19**)—7RK7	2	−8.6	1.60
(**20**)—2Y9X	1	−8.7	1.30	(**20**)—7RK7	1	−8.6	1.49

**Table 4:** The detail interaction of the most active phytocompounds of the *T. erubescens* EA extract with mushroom tyrosinase (2Y9X and 7RK7) at binding site 1 via the docking simulation.

Symbol of Ligand–Protein	Number (Type) of Linkages	Amino Acids of Protein 2Y9X Interacting with the Ligands [Distance (Å)/E (kcal/mol)/Linkage Type]
Interacting with protein 7RK7		
(**4**)—7RK7	2 H-donor	**Asp77** (2.96/−3.7/H-donor); **Thr73** (2.83/−1.2/H-donor)
(**5**)—7RK7	1 H-donor, 1 H-acceptor	**Ile106** (3.16/−0.7/H-donor); **Trp147** (3.19/−1.1/H-acceptor)
(**9**)—7RK7	1 H-donor, 2 H-acceptor	**Asp77** (2.83/−0.7/H-donor); **Arg97** (2.95/−0.6/H-acceptor); **Arg97** (2.82/−1.6/H-acceptor)
Interacting with protein 2Y9X		
(**2**)—2Y9X	1 H-donor, 1 pi-H	**Asp60** (3.17/−0.7/H-donor); **Glu97** (4.58/−0.5/pi-H)
(**4**)—2Y9X	2 H-donor	**Tyr62** (3.11/−0.9/H-donor); **Gln72** (2.92/−1.9/H-donor)
(**5**)—2Y9X	3 H-donor	**Gln72** (2.86/−2.1/H-donor); **Glu97** (2.86/−1.2/H-donor); **Asp60** (2.76/−5.0/H-donor)
(**6**)—2Y9X	3 H-donor	**Ile96** (3.06/−1.2/H-donor); **Gly326** (3.23/−0.6/H-donor); **Glu340** (3.19/−0.7/H-donor)
(**7**)—2Y9X	1 H-donor, 2 H-acceptor, 1 pi-H	**Asp60** (3.05/−0.5/H-donor); **Lys5** (2.83/−4.3/H-acceptor); **Lys5** (2.92/−3.2/H-acceptor); **Gln74** (4.50/−0.6/pi-H)
(**8**)—2Y9X	1 H-donor, 1 H-acceptor	**Asp60** (3.05/−1.0/H-donor); **Leu75** (3.18/−2.4/H-acceptor)

**Table 5:** Drug-likeness properties of phytocompounds (**1**–**19**) from *T. erubescens* EA extract and kojic acid (compound **20**), based on Lipinski’s Rule of Five.

	Lipinski’s Rule of Five				
Compd.	Mass	H-Bond Donor	H-Bond Acceptors	logP	Molar Refractivity
**1**	169	3	5	−0.8331	35.766895
**2**	290	5	6	1.5461	72.622993
**3**	353	5	9	−1.980601	79.889977
**4**	458	8	11	2.233202	108.920845
**5**	442	7	10	2.527601	107.256042
**6**	432	7	10	−0.0655	103.53405
**7**	610	10	16	−1.878802	137.495483
**8**	302	5	7	2.0109	74.050476
**9**	270	3	5	2.4196	70.813889
**10**	352	3	4	2.303	90.462379
**11**	273	1	3	2.77732	79.403778
**12**	300	4	7	1.3121	88.268875
**13**	206	2	1	1.6213	61.934883
**14**	270	0	2	5.6407	82.327972
**15**	164	0	0	3.656799	54.935986
**16**	164	0	0	3.782299	55.895985
**17**	172	2	2	1.2694	48.713585
**18**	214	0	4	1.6473	55.293983
**19**	352	3	6	2.796819	99.579773
**20**	142	2	4	−0.17871	32.389095
Lipinski’s rules	≤500	≤5	≤10	≤5	40–130

## Data Availability

All relevant data are contained within the paper. The data analyzed in this study are available from the corresponding authors upon reasonable request.

## Supplementary Materials

The following supporting information can be downloaded at https://www.mdpi.com/article/10.3390/life15070995/s1: Tables S1 and S2; Figures S1–S12.

## Appendix B

**Table A1:** The sizes and residues in these binding sites on protein 2Y9X.

Color	Site	Size	Residues
	1	184	1:(SER2 LYS5 LYS70 GLN72 PRO73 GLN74 LEU75 HIS76 TYR78 TYR82 THR324 MET325 GLY326 LEU327 ILE328 PRO338 GLU340 TYR343 GLN347 ASP348 PRO349)2:(ASP60 GLY61 TYR62 GLN90 LYS93 SER95 ILE96 GLU97 TYR98 PHE105 VAL109 PRO110 ARG111 GLU112 GLY113 GLY114)
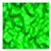
	2	47	1:(VAL27 LYS28 ASN29 ASP30 LYS31 PHE33 THR34 GLN153 ASP157 GLN159 VAL160 GLU161 ILE162 THR163 LYS169 GLU171)
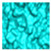
	3	22	1:(MET8 PRO9 VAL11 GLY12 ILE13 PRO14 GLN103 THR104 TRP106 GLU107)
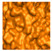
	4	52	1:(GLN307 THR308 ASN310 TYR311 ASP312 VAL313 TYR314 GLU356 ASP357 TRP358 LYS376 LYS379 SER380)
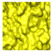

**Table A2:** The sizes and residues in these binding sites on protein 7RK7.

Color	Site	Size	Residues
	1	331	3:(ILE15 GLN45 PHE46 PRO47 SER48 GLN49 LEU87 SER88 ASP89 THR90 VAL92 TYR94 LYS112 LYS115 SER117 VAL118 LYS119 PRO120 SER150 ASP166 LYS167 CYS168 VAL169 ASN180)4:(HIS14 ARG40 GLN41 ASP42 PRO43 GLY44 HIS45 GLY46 ARG48 VAL64 ASP66 GLY67 TYR68 THR86 SER87 SER88 GLN89 THR90 SER91 VAL92 PHE94 TRP119 LEU120 THR121 VAL122 PHE160 ASP162 HIS163 VAL164 GLU165 LEU166 SER167 TRP168 GLU174 VAL175 HIS176 VAL179 CYS180 THR181 ASP182 PRO183 GLN184 PRO185 ASN193 ASP194 TYR197 LEU199 SER201)
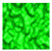
	2	219	1:(MET5 TYR7 PHE9 ALA24 MET45 TYR59 GLU63 LYS66 VAL67 ALA69 HIS70 THR73 ASP77 THR80 LEU81 TYR84 ARG97 TYR99 HIS114 TYR116 TYR123 THR142 THR143 LYS146 TRP147 ALA150 VAL152 GLN155 LEU156 TYR159 THR163 TRP167 TYR171)3:(ASN37 TYR39 LEU99 ASN100 TYR101 GLY102 GLN105)4:(LEU105 ILE106 PHE107 PRO108)
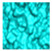
	3	151	1:(ARG6 PHE8 TYR27 ASP29 ASP30 THR31 GLN32 ARG48 PRO210 ALA211 GLU212 GLU232 THR233 ARG234 PRO235 GLY237 GLY239 PHE241)2:(TYR27 SER29 HIS52 SER53 ASP54 SER56 PHE57 SER58 LYS59 TYR64 LEU65 LEU66 TYR68)
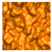
	4	59	1:(HIS188 TRP204 LEU206 VAL231 ARG234 GLN242 LYS243 TRP244)2:(GLN9 VAL10 TYR11 SER12 HIS14 PRO15 ALA16 GLU17 TRP96 ARG98 ASP99 MET100)
