# Supplementary material for: Novel and Potential Photoprotective and Tyrosinase Inhibitory Effects of Tetrastigma erubescens Extracts: Evidence from In Vitro Assays and Computational Approach
Source: Life (Basel). 2025 Jun 22;15(7):995. doi: 10.3390/life15070995 (PMC12300742; doi:10.3390/life15070995)
Supplement: Supplementary file 1 [file life-15-00995-s001.zip › life-3581402-supplementary.pdf]

Supplementary section

Table S1. The data prediction of pharmacokinetic properties and toxicity of phytochemicals (**1-10**) detected from the ethyl acetate extract of *T. erubescens*.

	Compounds									
**Properties**	**1**	**2**	**3**	**4**	**5**	**6**	**7**	**8**	**9**	**10**
**Absorption**										
Water solubility(1)	-2.56	-3.117	-2.449	-2.894	-2.892	-2.845	-2.892	-2.925	-2.892	-3.87
$CaCO_2$ permeability(2)	-0.081	-0.283	-0.84	-1.521	-0.949	-0.956	-0.949	-0.229	-0.949	1.023
Intestinal absorption (human)(3)	43.374	68.829	36.377	47.395	23.446	46.695	23.446	77.207	23.446	97.29
Skin permeability(4)	-2.735	-2.735	-2.735	-2.735	-2.735	-2.735	-2.735	-2.735	-2.735	-4.222
P-glycoprotein substrate(5)	No	Yes	Yes	Yes	Yes	Yes	Yes	Yes	Yes	Yes
P-glycoprotein I inhibitor(5)	No	No	No	No	No	No	No	No	No	No
P-glycoprotein II inhibitor(5)	No	No	No	Yes	No	No	No	No	No	No
**Distribution**										
VDss (human)(6)	-1.855	1.027	0.581	0.806	1.663	1.071	1.663	1.559	1.663	0.096
Fraction unbound (human)(6)	0.617	0.235	0.658	0.215	0.187	0.242	0.187	0.206	0.187	0.458
BBB permeability(7)	-1.102	-1.054	-1.407	-2.184	-1.899	-1.449	-1.899	-1.098	-1.899	-0.641
CNS permeability(8)	-3.74	-3.298	-3.856	-3.96	-5.178	-3.834	-5.178	-3.065	-5.178	-3.022
**Metabolism**										
CYP2D6 substrate(5)	No	No	No	No	No	No	No	No	No	No
CYP3A4 substrate(5)	No	No	No	No	No	No	No	No	No	No
CYP1A2 inhibitor(5)	No	No	No	No	No	No	No	Yes	No	No
CYP2C19 inhibitor(5)	No	No	No	No	No	No	No	No	No	No
CYP2C9 inhibitor(5)	No	No	No	No	No	No	No	No	No	No
CYP2D6 inhibitor(5)	No	No	No	No	No	No	No	No	No	No
CYP3A4 inhibitior(5)	No	No	No	Yes	No	No	No	No	No	No
**Excretion**										
Total clearance(9)	0.518	0.183	0.307	0.292	-0.369	0.444	-0.369	0.407	-0.369	0.775
Renal OCT2 substrate(5)	No	No	No	No	No	No	No	No	No	No
**Toxicity**										
AMES toxicity(5)	No	No	No	No	No	No	No	No	No	No
Max. tolerated dose (human)(10)	0.7	0.438	-0.134	0.441	0.452	0.577	0.452	0.499	0.452	0.075
hERG I inhibitor(5)	No	No	No	No	No	No	No	No	No	No
hERG II inhibitor(5)	No	No	No	Yes	Yes	No	Yes	No	Yes	No
Oral rat acute toxicity (LD50)(11)	2.218	2.428	1.973	2.522	2.491	2.595	2.491	2.471	2.491	2.107
Oral rat chronic toxicity(12)	3.06	2.5	2.982	3.065	3.673	4.635	3.673	2.612	3.673	1.809
Hepatotoxicity(5)	No	No	No	No	No	No	No	No	No	No
Skin sensitization(5)	No	No	No	No	No	No	No	No	No	No
T.Pyriformis toxicity(13)	0.285	0.347	0.285	0.285	0.285	0.285	0.285	0.288	0.285	0.325
Minnow toxicity (14)	3.188	3.585	5.741	7.713	7.677	4.897	7.677	3.721	7.677	3.115

Unit: (1) log $mol.L^{-1}$; (2) log Papp (10^{-6} $cm.s^{-1}$); (3) %; (4) log Kp; (5) Yes/No; (6) log $L.kg^{-1}$; (7) log BB; (8) log PS; (9) log $mL.min^{-1}.kg^{-1}$; (10) log $mg.kg^{-1}.day^{-1}$; (11) $mol.kg^{-1}$; (12) log $mg.kg^{-1}$_$bw.day^{-1}$; (13) log $\mu g.L^{-1}$; (14) log mM.

Table S2. The data prediction of pharmacokinetic properties and toxicity of phytochemicals (**11-19**) detected from the ethyl acetate extract of *T. erubescens* and kojic acid (**20**).

	Compounds									
**Properties**	**1**	**2**	**3**	**4**	**5**	**6**	**7**	**8**	**9**	**10**
**Absorption**										
Water solubility(1)	-3.988	-2.901	-2.795	-6.927	-5.224	-5.651	-1.468	-1.567	-3.435	-2.311
$CaCO_2$ permeability(2)	1.351	1.331	1.64	1.6	1.414	1.401	1.485	1.271	0.908	1.5
Intestinal absorption (human)(3)	94.452	92.644	93.401	92.335	96.219	95.175	76.806	97.718	89.412	94.491
Skin permeability(4)	-2.708	-2.735	-2.384	-2.595	-1.128	-1.009	-2.713	-2.868	-2.77	-2.167
P-glycoprotein substrate(5)	No	No	Yes	No	No	No	No	No	Yes	No
P-glycoprotein I inhibitor(5)	No	No	No	No	No	No	No	No	Yes	No
P-glycoprotein II inhibitor(5)	No	No	No	No	No	No	No	No	Yes	No
**Distribution**										
VDss (human)(6)	0.259	0.549	0.727	0.334	0.521	0.475	0.04	-0.021	-0.497	0.218
Fraction unbound (human)(6)	0.195	0.362	0.321	0.074	0.268	0.266	0.523	0.608	0.083	0.507
BBB permeability(7)	0.614	-1.02	0.358	0.749	0.806	0.852	-0.107	0.137	-1.393	0.568
CNS permeability(8)	-2.132	-3.016	-1.497	-1.678	-1.694	-1.632	-2.415	-3.249	-2.821	-2.473
**Metabolism**										
CYP2D6 substrate(5)	No	No	No	No	No	No	No	No	No	No
CYP3A4 substrate(5)	Yes	No	No	Yes	No	No	No	No	No	No
CYP1A2 inhibitor(5)	Yes	Yes	No	Yes	No	No	No	No	Yes	No
CYP2C19 inhibitor(5)	Yes	No	No	No	No	No	No	No	Yes	No
CYP2C9 inhibitor(5)	No	No	No	No	No	No	No	No	No	No
CYP2D6 inhibitor(5)	No	No	Yes	No	No	No	No	No	No	No
CYP3A4 inhibitior(5)	No	No	No	No	No	No	No	No	No	No
**Excretion**										
Total clearance(9)	0.228	0.878	1.519	1.861	0.237	1.766	0.323	0.842	0.318	1.269
Renal OCT2 substrate(5)	No	No	No	No	No	No	No	No	No	No
**Toxicity**										
AMES toxicity(5)	No	Yes	No	No	No	No	No	Yes	No	No
Max. tolerated dose (human)(10)	0.542	1.266	0.204	0.178	0.357	0.354	0.545	0.505	0.487	0.858
hERG I inhibitor(5)	No	No	No	No	No	No	No	No	No	No
hERG II inhibitor(5)	No	Yes	No	No	No	No	No	No	No	No
Oral rat acute toxicity (LD50)(11)	2.217	2.037	2.369	1.635	1.545	1.406	1.608	2.314	2.701	1.822
Oral rat chronic toxicity(12)	1.872	0.884	1.894	2.998	2.368	2.375	2.685	2.312	3.215	1.996
Hepatotoxicity(5)	No	Yes	Yes	No	No	No	No	No	Yes	No
Skin sensitization(5)	No	No	Yes	Yes	No	Yes	Yes	Yes	No	Yes
T.Pyriformis toxicity(13)	1.096	0.285	1.244	1.935	1.223	1.663	-0.182	-0.487	0.312	0.195
Minnow toxicity (14)	0.211	2.001	0.154	-1.373	0.45	0.162	1.879	1.578	2.407	1.484

Unit: (1) log mol.L^{-1}; (2) log Papp (10^{-6} cm.s^{-1}); (3) %; (4) log Kp; (5) Yes/No; (6) log L.kg^{-1}; (7) log BB; (8) log PS; (9) log mL.min^{-1}.kg^{-1}; (10) log mg.kg^{-1}.day^{-1}; (11) mol.kg^{-1}; (12) log mg.kg^{-1}_bw.day^{-1}; (13) log μg.L^{-1}; (14) log mM.

Chromatogram and Results

Injection Details

Injection Name:	**AQETYLok**	*Run Time (min):*	**26.00**
Vial Number:	**RB4**	*Injection Volume:*	**2.00**
Injection Type:	**Unknown**	*Channel:*	**UV_VIS_1**
Calibration Level:		*Wavelength:*	**265**
Instrument Method:	**MeOH-H3PO4 radian**	*Bandwidth:*	**n.a.**
Processing Method:	**Quantitative**	*Dilution Factor:*	**1.0000**
Injection Date/Time:	**21/Apr/24 17:12**	*Sample Weight:*	**1.0000**

Chromatogram

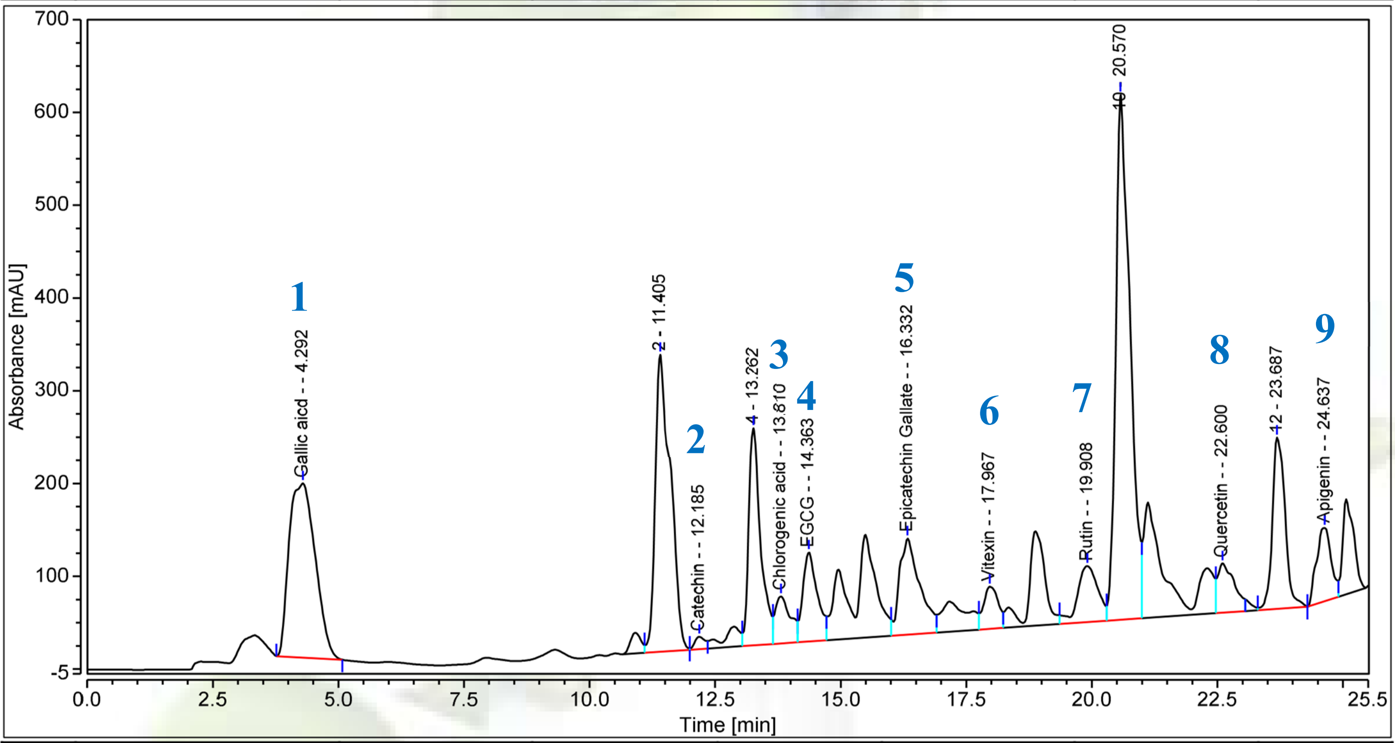

Integration Results

No.	Peak Name	Retention Time min	Area mAU*min	Height mAU	Relative Area %	Relative Height %	Amount
1	Gallic aicd	4.292	113.577	187.838	15.65	9.42	n.a.
2		11.405	112.579	320.092	15.52	16.06	n.a.
3	Catechin	12.185	3.111	13.269	0.43	0.67	n.a.
4		13.262	64.761	233.902	8.93	11.73	n.a.
5	Chlorogenic acid	13.810	17.840	50.702	2.46	2.54	n.a.
6	EGCG	14.363	32.233	96.010	4.44	4.82	n.a.
n.a.	Epicatechin	n.a.	n.a.	n.a.	n.a.	n.a.	n.a.
n.a.	Epicatechin	n.a.	n.a.	n.a.	n.a.	n.a.	n.a.
7	Epicatechin Gallate	16.332	48.536	103.600	6.69	5.20	n.a.
8	Vitexin	17.967	15.504	45.483	2.14	2.28	n.a.
n.a.	Salycylic acid	n.a.	n.a.	n.a.	n.a.	n.a.	n.a.
n.a.	Isovitexin	n.a.	n.a.	n.a.	n.a.	n.a.	n.a.
9	Rutin	19.908	28.606	60.440	3.94	3.03	n.a.
10		20.570	187.458	565.874	25.84	28.38	n.a.
n.a.	Myricetin	n.a.	n.a.	n.a.	n.a.	n.a.	n.a.
n.a.	unknow	n.a.	n.a.	n.a.	n.a.	n.a.	n.a.
11	Quercetin	22.600	20.325	53.183	2.80	2.67	n.a.
12		23.687	54.274	184.561	7.48	9.26	n.a.
n.a.	Kaempferol	n.a.	n.a.	n.a.	n.a.	n.a.	n.a.
13	Apigenin	24.637	26.726	78.748	3.68	3.95	n.a.
**Total:**			**725.530**	**1993.703**	**100.00**	**100.00**	

Figure S1. UHPLC spectrum of ethyl acetate extract from *T. erubescens*.

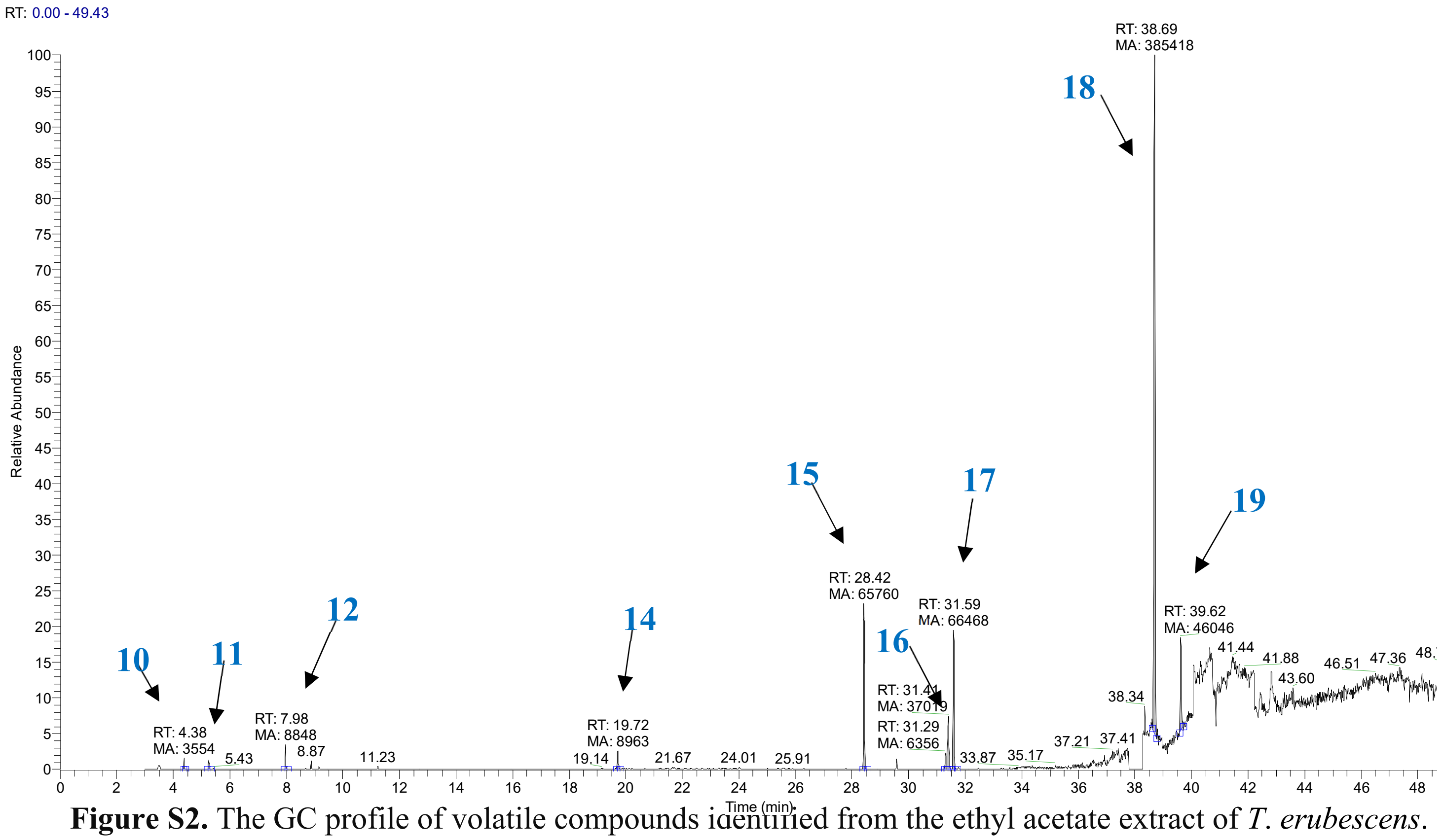

Figure S2. The GC profile of volatile compounds identified from the ethyl acetate extract of *T. erubescens*.

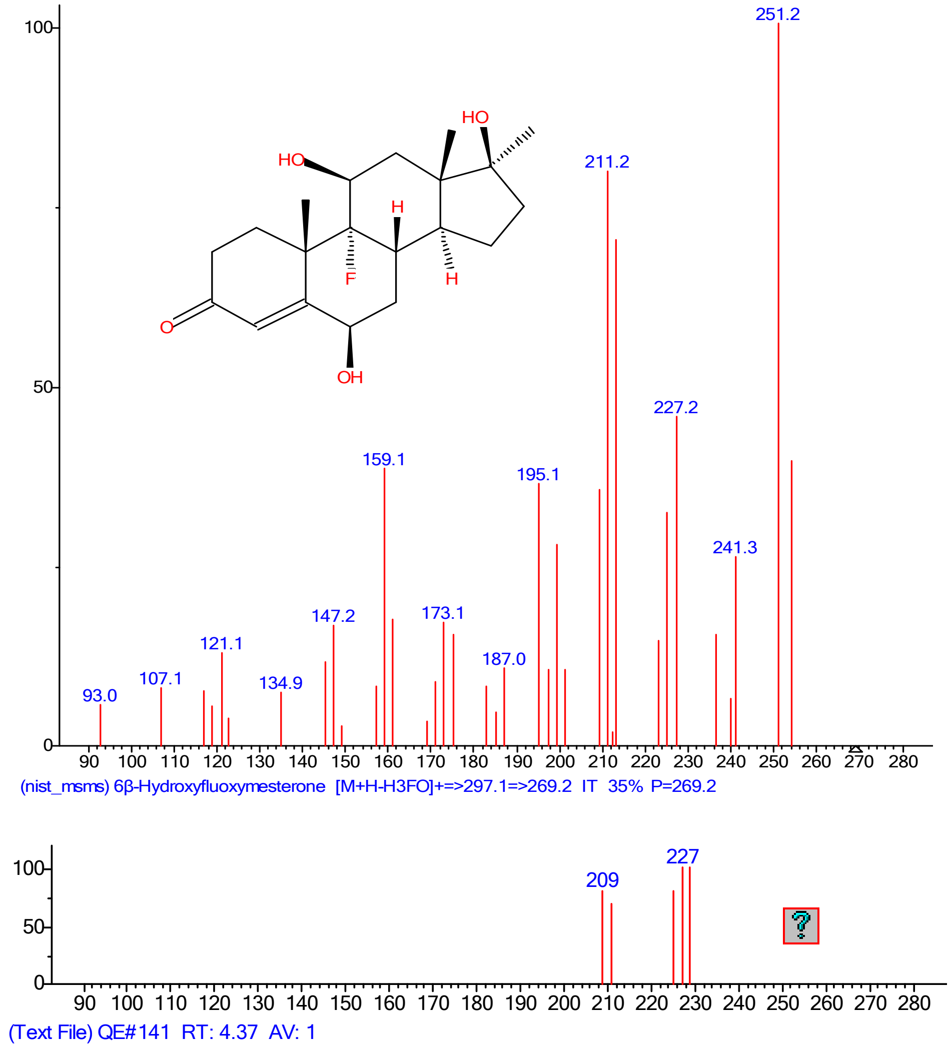

Figure S3. The mass spectra of 6*β*-Hydroxyfluoxymesterone (**10**).

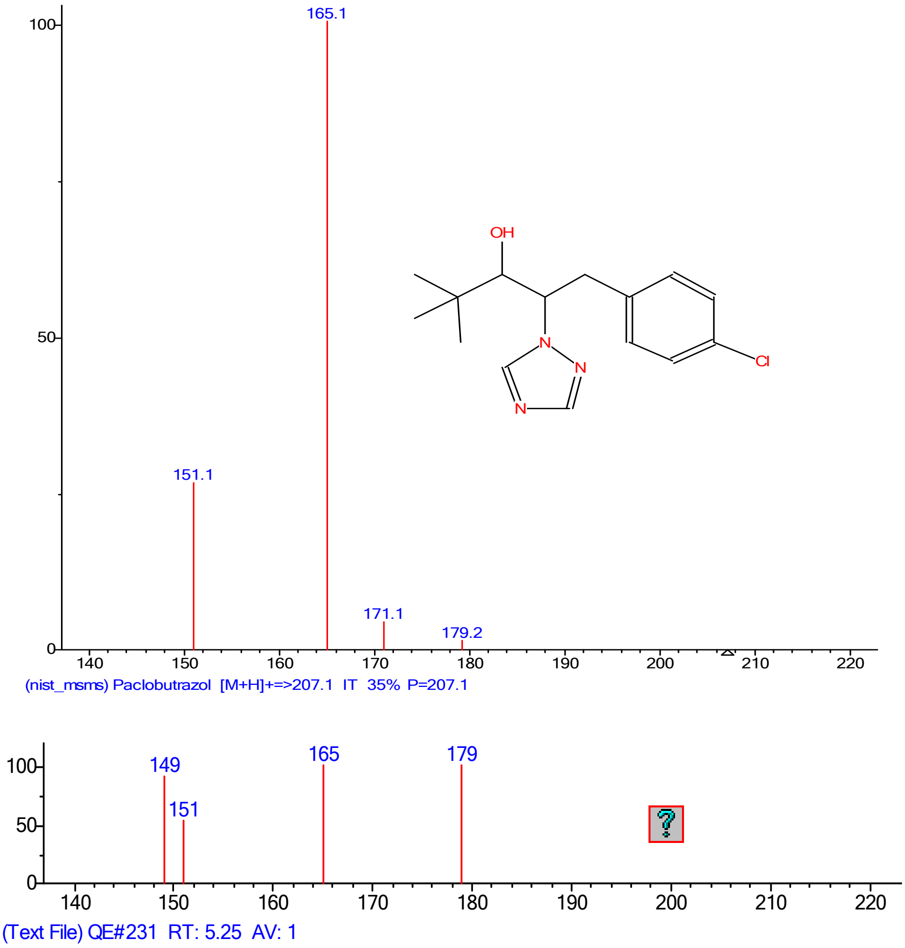

Figure S4. The mass spectra of Paclobutrazol (**11**).

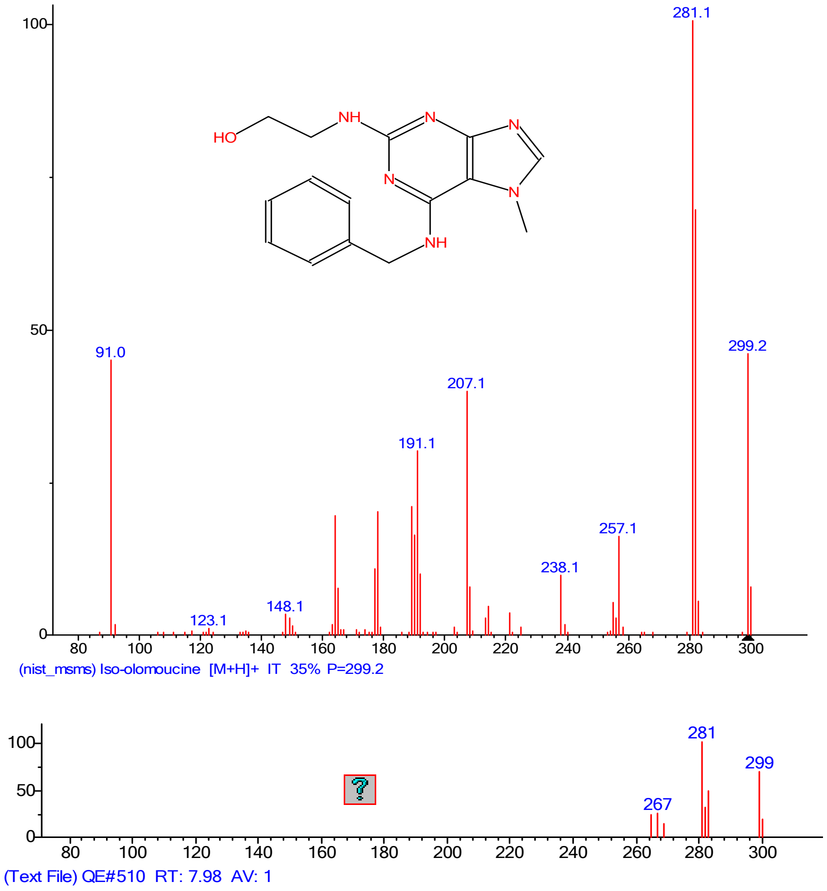

Figure S5. The mass spectra of Iso-olomoucine (**12**).

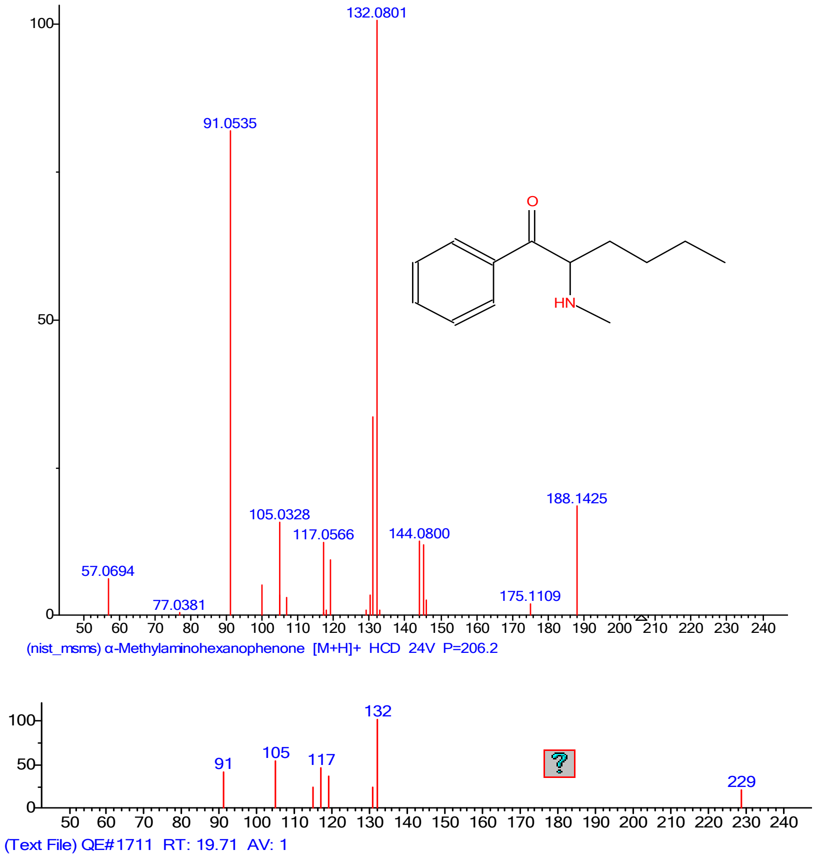

Figure S6. The mass spectra of *α*-Methylaminohexanophenone (**13**).

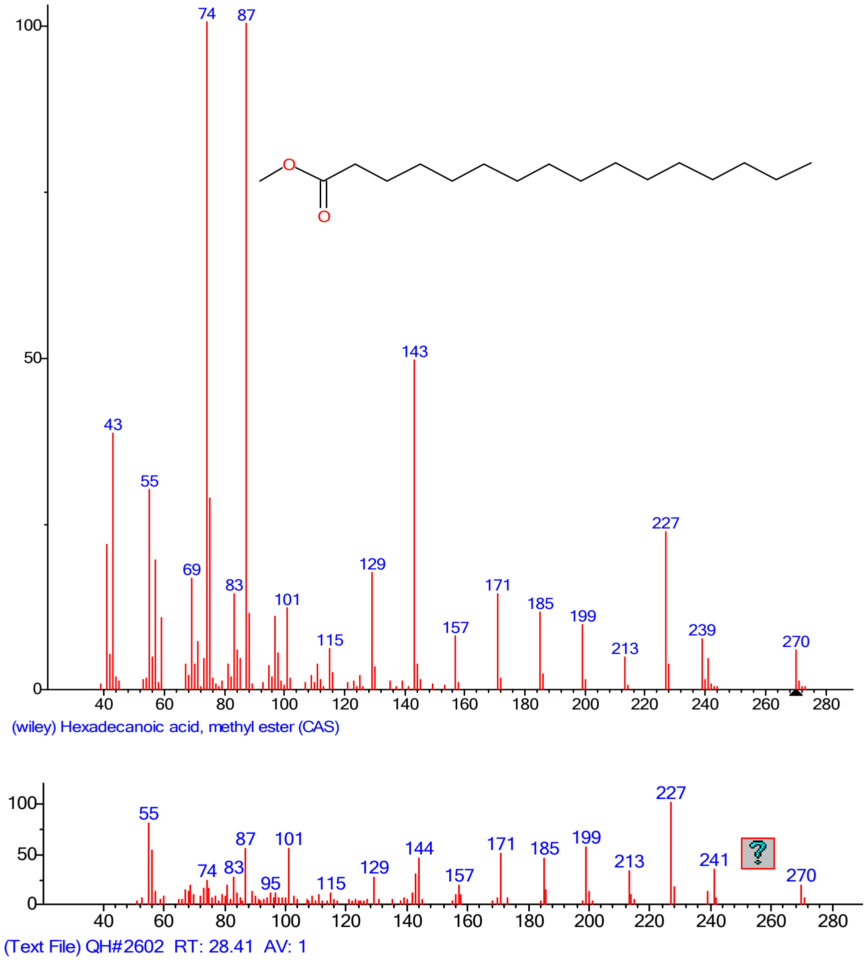

Figure S7. The mass spectra of Hexadecanoic acid, methyl ester (CAS) (**14**).

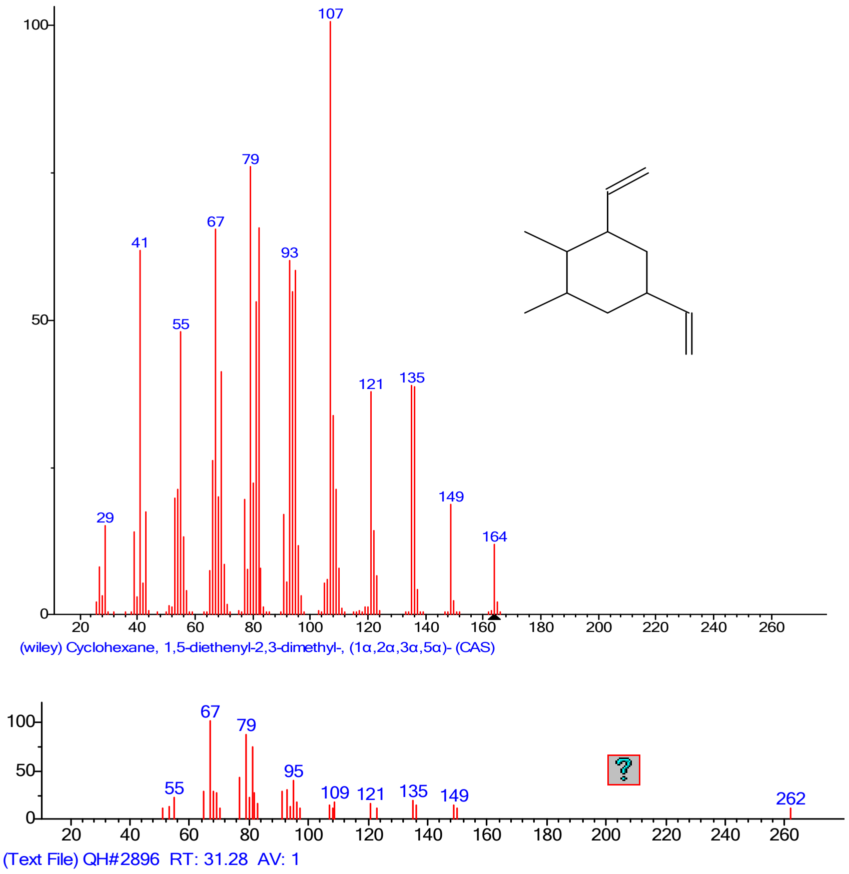

Figure S8. The mass spectra of Cyclohexane, 1,5-diethenyl-2,3-dimethyl-, (1α,2α,3α,5α)-(**15**).

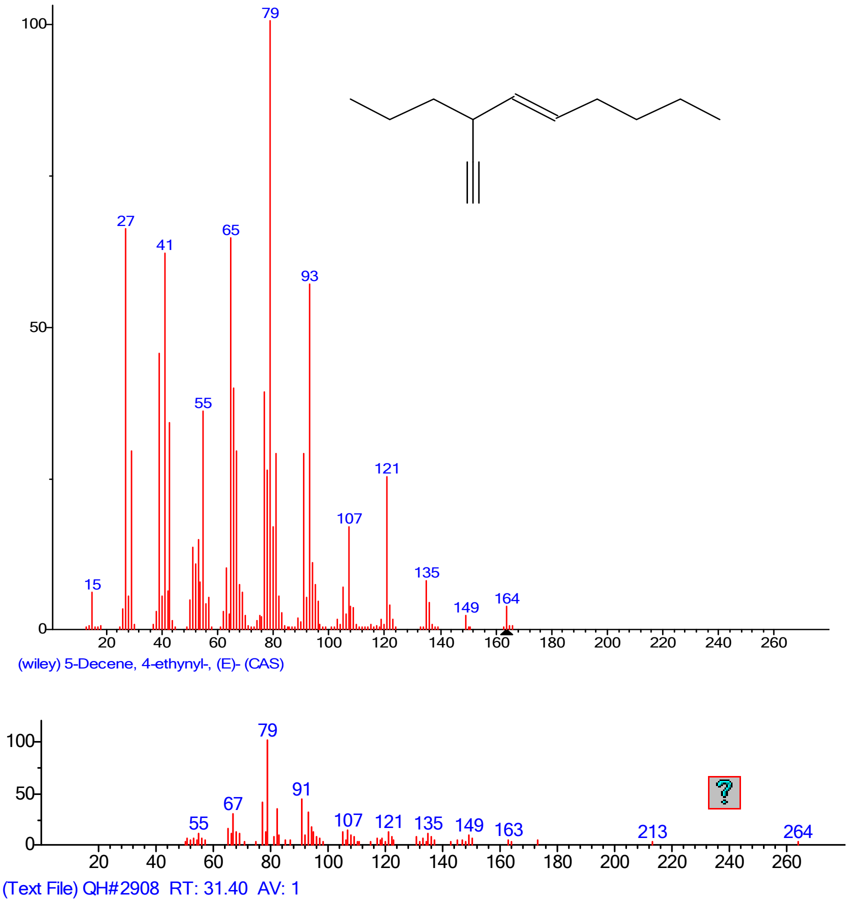

Figure S9. The mass spectra of 5-Decene, 4-ethynyl-, (E)- (CAS)(**16**).

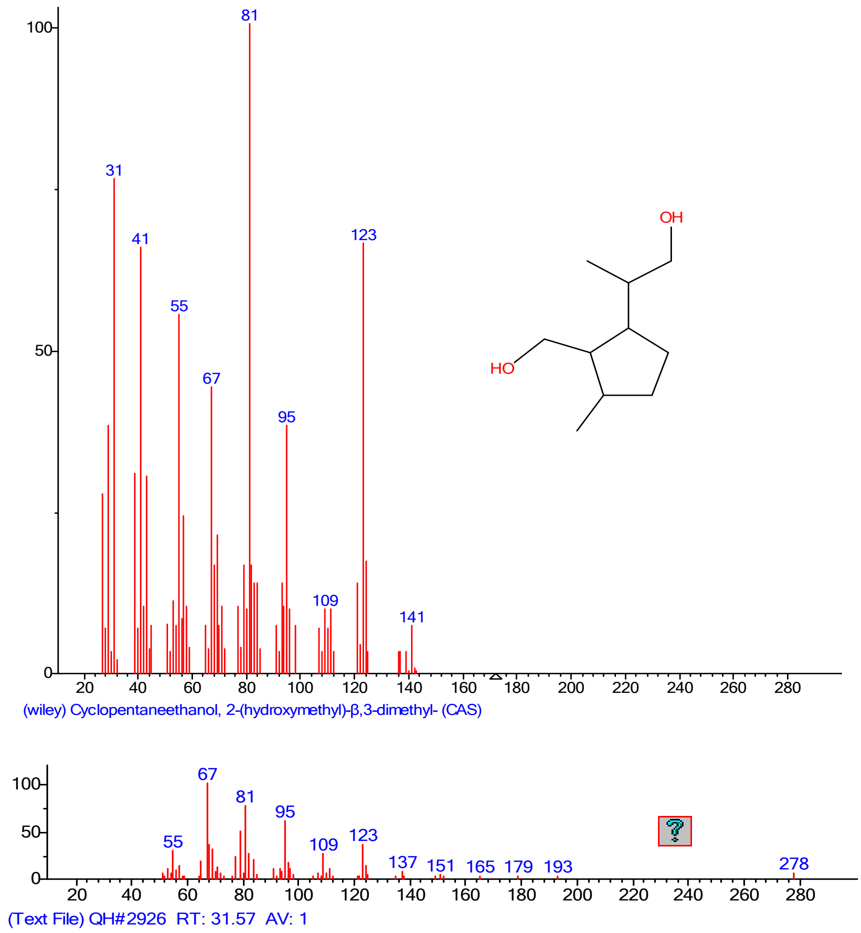

Figure S10. The mass spectra of Cyclopentaneethanol, 2-(hydroxymethyl)-β,3-dimethyl- (CAS)(**17**).

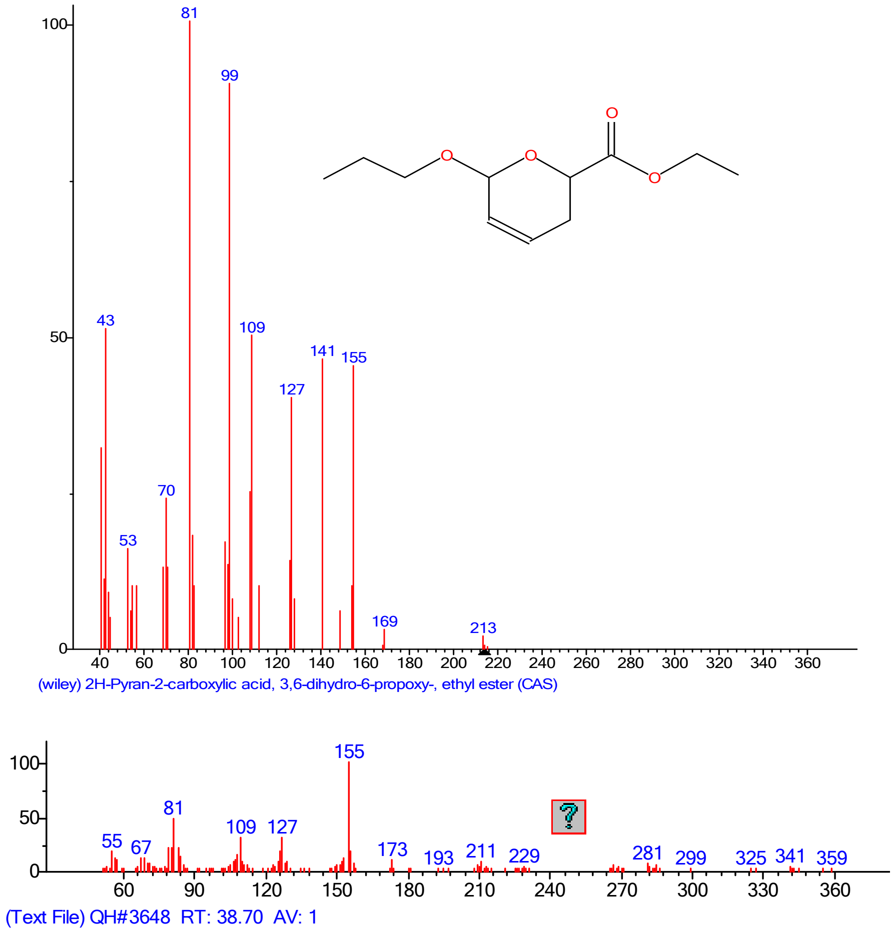

Figure S11. The mass spectra of 2H-Pyran-2-carboxylic acid, 3,6-dihydro-6-propoxy-, ethyl(**18**).

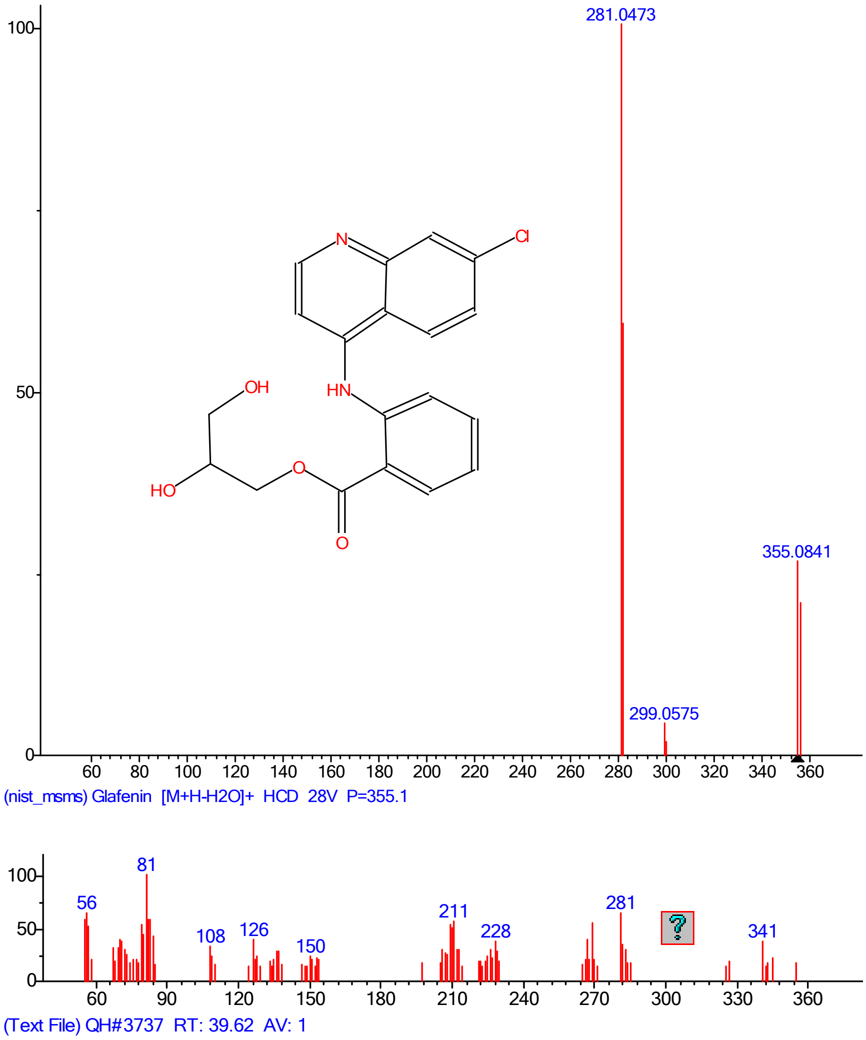

Figure S12. The mass spectra of Glafenin(**19**).
